# Treatment Results in the Differential Surgery of Intradural Extramedullary Schwannoma of 110 Cases

**DOI:** 10.1371/journal.pone.0063867

**Published:** 2013-05-27

**Authors:** Shaohui Zong, Gaofeng Zeng, Chunxiang Xiong, Bo Wei

**Affiliations:** 1 Department of Spine Osteopathia, the First Affiliated Hospital of Guangxi Medical University, Nanning, Guangxi, P.R. China; 2 College of Public Hygiene of Guangxi Medical University, Nanning, Guangxi, P.R. China; Universität München, Germany

## Abstract

**Study Design:**

A retrospective study of intradural extramedullary schwannoma.

**Objective:**

The purpose of this study was to compare treatment results in the differential surgery of intradural extramedullary schwannoma.

**Background:**

A reference guide to the surgical procedures available to treat intradural extramedullary schwannoma has not yet been established.

**Methods:**

The study retrospectively reviewed 110 patients: Group A: laminectomy+microscopic excision; Group B: hemilaminectomy+microscopic excision; Group C: laminectomy+microscopic excision+pedicle screw fixation. Researchers selected patients for this retrospective review by applying the following criteria: 1) back pain spread out from the tumor level, sensory and motor loss; 2) treatment by surgery; 3) clinical diagnosis made by physical examination, magnetic resonance imaging (MRI), and pathology; 4) a minimum clinical and radiologic follow-up of 12 months. The clinical outcomes were assessed by comparing the Visual Analogue Pain Scores (VAS) and the Japanese Orthopedic Association Scores (JOA score). The study also performed a cost-effectiveness analysis.

**Results:**

Cervical vertebrae: The estimated blood loss in Group B was significantly less than in Group C (P<0.05) (Table 1). Thoracic vertebrae: The duration of hospital stay and estimated blood loss in Group A was significantly less than in Group C (P<0.05) (Table 2, 3). Lumbar vertebrae: The resection rate in Group C was significantly higher than in Group A and Group B (P<0.05) (Table 4). Treatment in Group B was the least expensive, and therefore, the most cost-effective.

**Conclusion:**

In the case of appropriate surgical indications, the study suggests that hemilaminectomy+microscopic excision is advantageous in the removal of cervical schwannoma, and that laminectomy+microscopic excision is advantageous in the removal of thoracic schwannoma; lumbar intradural extramedullary schwannoma can be managed by laminectomy+microscopic excision+pedicle screw fixation.

## Introduction

Schwannoma is a neurogenic tumor originating from the Schwann cell. Intradural extramedullary schwannoma is one of the most common tumors of the spine [1]. The two main symptoms of intradural extramedullary schwannoma are radiculopathy and neurogenic claudication. When intradural extramedullary schwannoma grows, the effect is the compression of the spinal cord that usually causes worsening sensory and motor loss and back pain spreading out from the tumor level. Physical examination will reveal abnormal sensation below the level of the tumor, motor limitation, the Babinski sign, the Hoffman sign for cervical lesions, spasticity, bowel and bladder disorders, and hyperreflexia [2]. Due to the clinical symptoms and the imaging characteristics of intradural extramedullary schwannoma, it may be misdiagnosed as a prolapsed intervertebral disc or spinal canal stenosis. The best treatment for intradural extramedullary schwannoma is surgery.

The conservative therapy for intradural extramedullary schwannoma is limited with lasting effects noted in only a small number of cases [3]. Surgical intervention should be used when conservative therapy fails or in cases of initial signs of sensory and motor loss [4], [5]. The selection of the appropriate surgical procedure remains difficult and controversial. There are many surgical procedures, and all are based on the principles of decompression and complete tumor resection. These different surgical procedures have many good, fair, and poor consequences and each has specific complications. The improvement of symptoms and the complete resection of a tumor during an operation are particularly important [6], [7]. However, a reference guide to the surgical procedures available to treat intradural extramedullary schwannoma has not yet been established.

This study reviewed a series of 110 consecutive patients with intradural extramedullary schwannoma. The purpose of this retrospective, multicenter study was to compare treatment results in the differential surgery of intradural extramedullary schwannoma. In doing so, the study also addressed various surgical options for the treatment of intradural extramedullary schwannoma.

## Materials and Methods

### Ethics Statement

All study procedures were reviewed and approved by the Institutional Ethics Review Board at the First Affiliated Hospital of Guangxi Medical University and conducted according to the principles expressed in the Declaration of Helsinki. Informed consent was exempted by the board due to the retrospective nature of this research.

### Patient Characteristics

The current study retrospectively reviewed 110 patients who underwent surgery for intradural extramedullary schwannoma. Data were collected from the departments of surgery of six hospitals in the south of China over a period of 10 years (2001–2011). The average patient age was 46.7 years (23–75 years), and there were 52 men and 58 women. Mean symptom duration before surgery was 12.4 months (3–24 months). Patients were observed for an average of 34.6 months (12–78 months) (Table 5). Researchers selected patients for this retrospective review by applying the following criteria: 1) back pain spread out from the tumor level, sensory and motor loss; 2) treatment by surgery; 3) clinical diagnosis made by physical examination, magnetic resonance imaging (MRI), and pathology; 4) a minimum clinical and radiologic follow-up of 12 months. The spinal levels of intradural extramedullary schwannoma were cervical vertebra in 36 cases, thoracic vertebra in 37 cases, and lumbar vertebra in 37 cases.

### Surgical Methods

Six senior surgeons associated with the various hospitals performed all operations. The patients were divided into three groups according to the type of differential surgery: Group A: laminectomy+microscopic excision, 34 cases; Group B: hemilaminectomy+microscopic excision, 37 cases; Group C: laminectomy+microscopic excision+pedicle screw fixation, 39 cases.

### Preoperative and Postoperative Outcome Evaluation

The three groups were compared according to the levels of the tumor. Before surgery, the general health status of each patient was evaluated. After-surgery resection rates, operative times, blood losses, surgical complications, days spent in hospital, and total costs of hospitalization were evaluated. Before and after surgery, all patients completed the Visual Analogue Pain Score (VAS) for back pain [8]. Changes in neurological status were evaluated by using the Japanese Orthopedic Association scores (JOA scores) for cervical vertebra (JOA-C) [9], thoracic vertebrae (JOA-T) [10], and lumbar vertebrae (JOA-L) [11] both before surgery and during the final follow-up. The differences between the preoperative and final scores were evaluated: a full score on JOA-C was 17 points; a full score on JOA-T was 29 points; a full score on JOA-L was 11 points. The JOA score was determined via direct questioning to assess clinical signs, subjective symptoms, and the restriction of activities of daily living.

### Cost-effectiveness Analysis

The cost-effectiveness analysis was based on operative time, hospital stay, and internal fixation device cost [12]. The total costs of hospitalization included internal fixation device cost, drug cost, hospital bed cost, and surgery cost. Because all the patients received similar treatment, the cost of inpatient and outpatient physiotherapy was excluded. The cost of an internal fixation device complication was excluded, as none of the patients needed reoperation during the follow-up period. Because the patients were from different socioeconomic backgrounds, an accurate estimation of work loss-related costs was not possible and this item was excluded from the cost analysis.

### Statistical Analysis

All data are presented as the mean ± SEM. The statistical analysis was performed using SPSS 16.0 (SPSS, Inc., Chicago, IL, USA). Enumeration data were tested by chi-squared test. Measurement data were tested by analysis of variance and t test. A p<0.05 (two-tailed) was considered statistically significant.

## Results

### Baseline and Characteristics of Patients

Table 5 shows the descriptive characteristics of the three groups. There was no significant difference in age or gender ratio. There was no significant difference in the baseline data, including the course of the disease and the tumor size among the three groups (P>0.05) (Table 5).

### Clinical Results

Cervical vertebrae: There were no significant differences in operating times, durations of hospital stay, or resection rates among the three groups (P>0.05). The estimated blood loss in Group B was significantly less than in Group C (P<0.05). There were no significant differences in preoperative JOA-C scores or VAS among the three groups. Postoperative JOA-C scores increased significantly in all three groups compared to pre-operative values (P<0.05). The postoperative VAS decreased significantly in all three groups compared to pre-operative values (P<0.05). However, there were no significant differences in the postoperative JOA-C scores or VAS among the three groups (P>0.05). The complication rate in Group C was higher than in the other two groups. Group C had the most complications. Three complications were observed in Group C, including one case of infection of the incision wound, one case of leakage of cerebrospinal fluid, and one case of extradural hematoma. There were no complications in group B (Table 1).

**Table 1:** Clinical outcomes of the levels of schwannoma at cervical vertebrae.

Groups	n	Estimated bloodloss (ml)	POSTJOA-C	PRE VAS	POST VAS
A	11	345.130±14.78	13.6±2.6	7.6±0.8	1.6±0.5
B	12	330.35±11.95	14.5±2.2	7.5±0.6	1.7±0.8
C	13	791.61±12.81	13.8±2.8	7.7±0.9	1.7±0.3

This table shows that the clinical outcomes of the levels of schwannoma at cervical vertebrae.

PRE: preoperative; POST: postoperative; JOA-C: Japanese Orthopedic Association scores (JOA scores) for cervical vertebra; VAS: Visual Analogue Pain Score.

Data are expressed as the mean ± standard deviation.

Thoracic vertebrae: There were no significant differences in operating times or resection rates among the three groups (P>0.05). The duration of hospital stay and estimated blood loss in Group A was significantly less than in Group C (P<0.05). There were no significant differences in preoperative JOA-T scores or VAS among the three groups. The postoperative JOA-T scores increased significantly compared to pre-operative values in all three groups (P<0.05). The postoperative VAS decreased significantly compared to pre-operative values in all three groups (P<0.05). However, there were no significant differences in the postoperative JOA-T scores or VAS among the three groups (P>0.05). The complication rate in Group C was higher than in the other three groups. Group C had the most complications. Three complications related to group C included two cases of infection of the incision wound, two cases of cerebrospinal fluid leakage, and one case of paraplegia. There was one complication in group A: a single case of cerebrospinal fluid leakage (Table 2, 3).

**Table 2:** Clinical outcomes of the levels of schwannoma at thoracic vertebrae.

Groups	n	Estimated bloodloss (ml)	Duration of hospitalstay (d)
A	12	331.10±132.56	20.1±5.41
B	12	344.04±204.85	22.67±9.26
C	13	781.50±770.09	26.73±16.72

This table shows that the clinical outcomes of the levels of schwannoma at thoracic vertebrae.

Data are expressed as the mean ± standard deviation.

**Table 3:** Clinical outcomes of the levels of schwannoma at thoracic vertebrae.

Groups	n	PRE JOA-C	POST JOA-C	PRE VAS	POST VAS
A	12	4.3±1.5	9.0±2.4	7.4±0.5	1.7±0.2
B	12	4.2±1.5	9.3±3.1	7.7±0.2	1.6±0.7
C	13	4.9±2.1	9.2±5.7	7.9±0.4	1.6±0.6

This table shows that the clinical outcomes of the levels of schwannoma at thoracic vertebrae.

PRE: preoperative; POST: postoperative; JOA-C: Japanese Orthopedic Association scores (JOA scores) for cervical vertebra; VAS: Visual Analogue Pain Score.

Data are expressed as the mean ± standard deviation.

Lumbar vertebrae: There were no significant differences in operating times, estimated blood loss, or durations of hospital stay among the three groups (P>0.05). The resection rate in Group C was significantly higher than in Group A and Group B (P<0.05). There were no significant differences in preoperative JOA-L scores or VAS among the three groups. The postoperative JOA-L scores increased significantly compared to pre-operative values in all three groups (P<0.05). The postoperative VAS decreased significantly in the three groups compared to pre-operative values (P<0.05). However, there were no significant differences in the postoperative JOA-L scores or VAS among the three groups (P>0.05). There were no significant differences in complication rates among the three groups (Table 4).

**Table 4:** Clinical outcomes of the levels of schwannoma at lumbar vertebrae.

Groups	n	Resectionrate (%)	PREJOA-C	POSTJOA-C	PRE VAS	POST VAS
A	11	70.49	12.4±2.5	21.3±1.6	7.2±0.7	1.7±0.4
B	13	70.27	12.6±2.4	22.4±2.1	7.0±0.9	1.7±0.4
C	13	82.23	12.4±2.5	21.5±3.6	7.1±0.8	1.7±0.6

This table shows that the clinical outcomes of the levels of schwannoma at lumbar vertebrae.

PRE: preoperative; POST: postoperative; JOA-C: Japanese Orthopedic Association scores (JOA scores) for cervical vertebra; VAS: Visual Analogue Pain Score.

Data are expressed as the mean ± standard deviation.

**Table 5:** Summary of baseline characteristics of intradural extramedullary schwannoma patients.

Groups	n	Age (year)	Male/Female	Course of disease (month)	Tumor size (cm^3^)
CV-A	11	62.17±4.56	2/3	9.24±2.81	8.92±2.91
CV-B	12	62.82±3.35	3/4	17.89±4.39	6.62±2.17
CV-C	13	62.91±5.42	1/1	10.63±5.95	13.09±3.89
TV-A	12	61.15±1.26	1/1	10.26±1.83	9.82±1.01
TV-B	12	62.84±2.37	4/3	15.19±2.19	6.92±2.07
TV-C	13	61.61±3.62	1/1	12.65±6.05	13.19±2.19
LV-A	11	60.61±1.28	3/4	11.56±3.73	10.11±2.01
LV-B	13	61.27±2.46	1/1	16.10±2.09	7.02±1.07
LV-C	13	60.24±2.73	4/5	11.75±2.95	14.19±3.12

This table showed that there was no significant difference among the three groups (Group A, Group B, Group C) in the basic characteristics (Age distribution, Gender distribution, Course of disease, and Tumor size).

CV: Cervical vertebra; TV: Thoracic vertebra; LV: Lumbar vertebra.

Data are expressed as the mean ± standard deviation.

### Cost-effectiveness Analysis

Cost effectiveness of the differential surgery of intradural extramedullary schwannoma was analyzed by calculating the total costs of hospitalization in all patients as shown in Table 6. The levels of schwannoma were cervical vertebrae, thoracic vertebrae, and lumbar vertebrae: the average total cost in Group B was significantly less than in the other two groups (P<0.05). The average total cost in Group C was significantly higher than in the other two groups (P<0.05) (Table 6).

**Table 6:** Analysis of cost effectiveness of the various surgical approaches.

Groups	Total cost	n	The average total cost
CV-A	¥207659.1	11	¥18878.10±479.39
CV-B	¥252464.32	12	¥12705.36±737.01
CV-C	¥427969.36	13	¥32920.72±236.08
TV-A	¥238009.2	12	¥19834.10±479.36
TV-B	¥161584.32	12	¥13465.36±537.04
TV-C	¥451370.66	13	¥34720.82±226.13
LV-A	¥229287.96	11	¥20844.36±274.35
LV-B	¥187967.13	13	¥14459.01±237.23
LV-C	¥478167.69	13	¥36782.13±626.63

This table shows that analysis of cost effectiveness of the various surgical approaches.

CV: Cervical vertebra; TV: Thoracic vertebra; LV: Lumbar vertebra.

Data are expressed as the mean ± standard deviation.

## Discussion

Conservative treatment for intradural extramedullary schwannoma is mostly ineffective, and surgical treatment is necessary. However, many authors [13], [14] have reported poor surgical outcomes, so that information about a surgical procedure of choice cannot be established. This article tries to assess the different surgical procedures used for intradural extramedullary schwannoma from a retrospective study to provide some benchmarks for the optimal surgical procedure.

In the present study, neurologic function was assessed before surgery and during the final follow-up using a scoring system proposed by the Japanese Orthopedic Association (JOA score) [15]. The postoperative JOA-C, JOA-T, and JOA-L scores were improved significantly in all three groups compared to pre-operative values (P<0.05). Regarding post-operative status, there were no significant differences in the postoperative JOA scores among the three groups. The VAS was a two-item numerical rating scale from 0 (no discomfort or pain) to 10 (unbearable pain) for back pain [16]. The present results at follow-up showed good improvement of VAS, but no significant differences among the groups. For most intradural extramedullary schwannoma patients, improvement of neurologic function and pain were observed post-operatively, which was thought to be the principal goal of the surgery for the patients [17]. Thus, the three surgical methods all appear to achieve improved quality of life, in patients with schwannoma of the cervical vertebra, the thoracic vertebra, and the lumbar vertebra.

In the present study, clinical outcomes of the removal of intradural extramedullary schwannoma by hemilaminectomy+microscopic excision (Group B) were re­viewed in 37 cases. Hemilaminectomy+microscopic excision would be optimal for tumors with clear borders, extramedullary, and extradu­ral tumors. Researchers noted several ­advantages of hemilaminectomy+microscopic excision in the removal of intradural extramedullary schwannoma. The estimated blood loss in Group B (hemilaminectomy+microscopic excision) was significantly less than in Group C among patients with cervical schwannoma (P<0.05). Researchers found that hemilaminectomy+microscopic excision decreased the amount of complications in patients with cervical schwannoma. Hemilaminectomy+microscopic excision is advantageous in the removal of cervical schwannoma. However, the hemilaminectomy approach provides a relatively narrow view of the spinal intracanalar regions [18]; so, large intradural extramedullary schwannomas that require resection are difficult to manage by hemilaminectomy+microscopic excision.

Conventional microscopic excision is a widely accepted method for the management of a variety of spinal tumors. However, the long incisions and extensive detachment of muscle from the spinal processes can result in ischemic necrosis and massive bleeding [19]. To reduce the likelihood of iatrogenic muscle injury and iatrogenic spinal cord injury, microscopic techniques have been developed. The concept of “microscopic techniques”, however, results not only in short incisions, but also in less estimated blood loss and in maximum therapeutic results [20]. In patients with thoracic schwannoma, results suggested that the laminectomy+microscopic excision (Group A) is superior to the laminectomy+microscopic excision+pedicle screw fixation (Group C) in terms of less estimated blood loss and a shorter hospital stay. Laminectomy+microscopic excision is advantageous in the removal of thoracic schwannoma.

One of the goals of treatment in intradural extramedullary schwannoma is the maintenance of vertebral column stability and obtaining spinal canal decompression, leading to early mobilization of the patient. Pedicle-screw fixation is a widely accepted method for the management of a variety of spinal tumors that require spinal stabilization. The importance of spinal stabilization has been emphasized in several reports [21], [22]. Pedicle-screw fixation allows immediate stable fixation, preserves motion segments, controls segmental motion in three dimensions, and provides a more stable construct [23]. This study retrospectively analyzed patients who were treated for intradural extramedullary schwannoma, using pedicle-screw fixation in patients with lumbar schwannoma. The results suggested that the resection rate in the laminectomy+microscopic excision+pedicle screw fixation group (Group C) was significantly higher than in the laminectomy+microscopic excision group (Group A) and the hemilaminectomy+microscopic excision group (Group B) (P<0.05). Laminectomy+microscopic excision+pedicle screw fixation are advantageous in removal of lumbar schwannoma.

One cannot use cost-effectiveness analysis to determine the “best” treatment for any particular disease. It is an analytical technique that involves combining many variables in a single model that allows systematic testing of the effect of each variable on outcomes [24]. In all three groups of patients with intradural extramedullary schwannoma, the total costs of hospitalization were considered in trying to determine the relative cost-effectiveness of the three surgical methods. The average total cost in the hemilaminectomy+microscopic excision group (Group B) was significantly less than in the other two groups in patients with cervical, thoracic, and lumbar schwannoma (P<0.05). The hemilaminectomy+microscopic excision is advantageous in the removal of cervical schwannoma, because hemilaminectomy+microscopic excision results in reduced hospital stay, less estimated blood loss, and reduced total costs of hospitalization in patients with cervical schwannoma. Because the costs of the internal fixation device must be taken into consideration, the average total cost in Group C was significantly higher than in the other three groups (P<0.05). The cost analysis was based on data from six hospitals, and therefore, may not be representative of costs at other hospitals. If post-discharge costs, outpatient physiotherapy, and home health aides were taken into consideration, the total cost of the procedures would be expected to increase. This study provides evidence on the degree of clinical improvement and an analysis of hospitalization costs only.

In this study, researchers found that clinical outcomes were satisfactory and comparable using all surgical methods. Hemilaminectomy+microscopic excision for patients with cervical intradural extramedullary schwannoma were the least expensive, and therefore, the most cost-effective approach. In the case of appropriate surgical indications, the study suggests that hemilaminectomy+microscopic excision is advantageous in the removal of cervical schwannoma, and that laminectomy+microscopic excision is advantageous in the removal of thoracic schwannoma; lumbar intradural extramedullary schwannoma can be managed by laminectomy +microscopic excision+pedicle screw fixation. The study provides some insight to help surgeons choose the most appropriate surgical method for intradural extramedullary schwannoma.
